# Novel Approaches for the 3D Printing of Collagen-Sourced Biomaterials Against Infectious and Cardiovascular Diseases

**DOI:** 10.3390/gels11090745

**Published:** 2025-09-16

**Authors:** Yugyung Lee, Chi H. Lee

**Affiliations:** 1 Department of Computer Science, School of Science and Engineering, University of Missouri-Kansas City, Kansas City, MO 64108, USA; leeyu@umkc.edu; 2 Division of Pharmacology, Toxicology and Pharmaceutics Sciences, University of Missouri-Kansas City, Kansas City, MO 64108, USA

**Keywords:** collagen, hydrogel, 3D printing, tissue engineering, infectious diseases

## Abstract

Collagen is a versatile material, and collagen in the human body strengthens the muscles and related organs, allowing good substances to be absorbed into the bloodstream while preventing the absorption of toxic substances. Thus, collagen has been broadly applied in regenerative medicine and tissue engineering. A comprehensive framework for various collagen products has been created by integrating collagen resources with additive components. The application of 3D-bioprinting technologies for designing physiological models further allows for the introduction of enhanced preclinical testing tools that can contribute to successful elucidation of the mechanisms behind host–pathogen interactions, and subsequent prevention and treatment of various diseases. In this review, novel strategies for the 3D-printing production of collagen-sourced biomedical devices, as well as diverse applications customized with advanced artificial intelligence (AI) technologies, were thoroughly examined. Ongoing challenges, including the inherent limitations in the mechanical weakness of collagen-based bioinks, such as printability and stability, along with cell viability and bioavailability, and advanced strategies addressing those challenges, were also reviewed. An integration of 3D printing with collagen as a bioink is enormously efficient in biomedical applications, demonstrating its great potential for clinical translation against infectious diseases, including cardiovascular diseases.

## 1. Introduction

An introduction of 3D additive manufacturing as well as advanced technology, including artificial intelligence (AI) and quantum computing (QC), allows us to develop such novel platforms as in vitro cell models, biomedical devices, biomimetic scaffolds, and the nano- and mesoscale formulations [1,2,3]. In particular, the application of 3D-bioprinting technologies for designing physiological models allows for the accelerated introduction of enhanced preclinical testing tools [4] that can contribute to the successful elucidation of the mechanisms behind host–pathogen interactions, and the subsequent prevention and treatment of various diseases [5,6].

Collagen, a naturally plentiful protein in the body, offers structure to skin, bones, tendons, muscles, and ligaments, but is considered an incomplete protein because it lacks some essential amino acids, including tryptophan [7]. Even though collagen was initially introduced as a cell growth matrix, the FDA approved collagen as a bone graft in 1991 [8]. Since then, collagen has established its prominence as an outstanding biomaterial, owing to its superb biocompatibility, ability to mimic the natural extracellular matrix (ECM), and dexterous ability to regulate the physiological cell processes [9,10]. As collagen and its composites resemble the structure and composition of specific tissues and organs, collagen has been subsequently applied in regenerative medicine and tissue engineering, including the design of cell models as well as the repair of both soft tissue (i.e., skin, cartilage, heart, and blood vessel) and hard tissue (i.e., bone, skull, teeth, and spine) [11].

**Figure 1:**
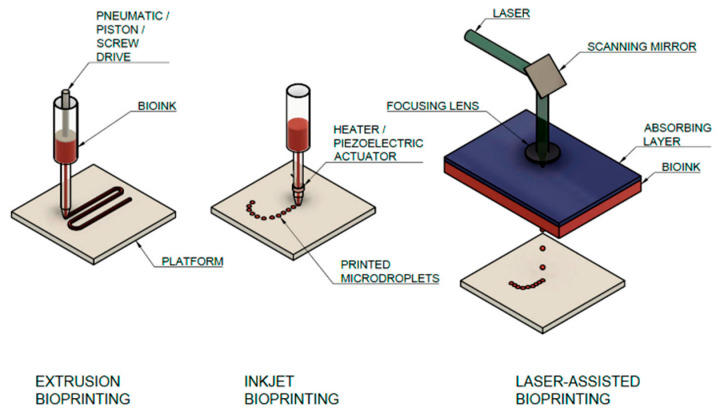
Main additive manufacturing technologies for collagen bioprinting, with a description of the main parts of the assemblies [16].

Numerous studies have explored collagen as a 3D-printing bioink in the treatment of defective organ cells [12,13]. The extrusion method, inkjet, and laser-assisted bioprinting are three distinct hydrogel-dispensing methods for creating 3D products, and each has unique mechanisms and applications, as shown in Figure 1. Also, 3D-printing techniques, such as stereolithography (SLA), digital light processing (DLP), and projection micro-stereolithography (PµSL) [14] utilize light to preserve liquid resin, hardening its layers based on the principle of two-photon absorption techniques [15].

Recent progress in AI technologies not only addresses existing issues stemming from biomedical applications of collagen (i.e., printability, stability, and cell viability, etc.), but also supports the tailoring of collagen materials in clinical applications. Collagen products manufactured and optimized via the integration of 3D printing and an AI-driven modeling process are enormously efficient in biomedical applications, demonstrating their great potential for clinical translation against infectious diseases, including cardiovascular diseases, where modulation of patient-specific pathological and genetic variables is integral.

In this review, novel strategies for the 3D printing of collagen-sourced biomaterials and their preparation methods were examined. Moreover, 3D-printing modalities, as well as diverse applications customized with advanced AI technologies against infectious and cardiovascular diseases, were thoroughly reviewed. Ongoing challenges, including the inherent limitations in printability, stability, and cell viability, the mechanical weakness of collagen-based bioinks, and advanced strategies addressing those challenges, were also discussed.

## 2. Preparation of Collagen and General Usage

### 2.1. Major Resources of Collagen

The successful development of collagen-based platforms for tissue engineering and regenerative medicine requires thorough analysis of multiple factors, such as the source of reliable raw materials, the application of efficient extraction techniques, the tailoring of physicochemical properties, and the incorporation of safe additive ingredients. The selection process is integral, as the functional outcome of collagen-based constructs not only includes the inherent biochemical features of collagen but also its compatibility with other biomaterials and its performance in biological environments. Designing such materials thus benefits from a discrete choice model, whose evaluation criteria include functionality, durability, biocompatibility, environmental impact, cost, and scalability of the manufacturing process [15].

Among those structural proteins in the body, Type I collagen plays the most prominent role in biomedical applications, as shown in Table 1. It is a fibril-forming protein composed of triple-helical units that spontaneously self-assemble into fibrils (length ≈ several micrometers, diameter ≈ 100 nm) [17,18]. These fibrils are further organized into collagen fibers [19,20], whose architecture varies depending on the tissue context. For example, fibers are randomly oriented in skin, whereas they display preferential, highly ordered alignment in tendons. The classification of collagen types reflects differences in the amino acid sequences of their polypeptide chains, which determine structural and functional variations [19].

Type I collagen provides both structural architecture and functional integrity to tissues. When extracted from highly organized tissues, such as bovine, porcine, or ovine tendons, it often retains partial lateral packing of fibrils despite the disruptive forces generated during extraction. By contrast, collagen isolated from randomly organized tissues, such as skin, does not preserve this ordered packing [23,24,28]. This distinction is critical—tendon-derived collagen, with its residual fibrous integrity, demonstrates enhanced resistance to mechanical stress and slower rates of enzymatic degradation compared with skin-derived collagen [23,29]. Consequently, tendon collagen—particularly equine (horse) collagen—has become one of the primary resources for biomedical applications, including surgical implants and regenerative scaffolds [24,25].

In recent years, fish-derived collagen has attracted attention as a potential alternative resource. Type I collagen extracted from fish skin or scales exhibits potential bioactivity, particularly in promoting tissue regeneration and wound healing [26]. However, fish collagen presents notable limitations—both fish- and mammal-derived collagens often suffer from low mechanical strength and limited antibacterial activity, which restrict their direct clinical utility [30]. Furthermore, the biological mechanisms by which fish collagen influences each cell type and supports tissue regeneration remain in its infancy. These limitations emphasize the importance of further systematic and multidisciplinary studies to fully assess the feasibility of fish collagen as a robust scaffold material.

### 2.2. Extraction Techniques for Collagen

The production of collagen for biomedical devices involves three critical stages, as follows: (1) extraction procedures, which include the choice of method, enzyme type and concentration, temperature, pH, exposure time, and homogenization approaches; (2) material optimization, encompassing fibrillogenesis, homogenization, and post-processing stabilization; and (3) manufacturing, where additive manufacturing techniques are applied under thoroughly controlled concentration, temperature, and pH conditions [26,30]. Among these stages, the extraction procedure is particularly crucial, as it determines not only the yield but also the physicochemical integrity and functions of collagen, thereby shaping all subsequent optimization and manufacturing processes.

Collagen can be isolated from mammalian tissues or by-products, such as the bones, tendons, feathers, hides, as well as the bones, scales, and swim bladders of fish [23,24,26,28,30], through a variety of extraction methods, including acid-soluble collagen (ASC), enzyme-soluble collagen (ESC), ultrasound-assisted extraction, deep eutectic solvent (DES) extraction, and supercritical fluid extraction (SFE) [26]. Each method targets specific structural components of collagen and varies in efficiency, scalability, and impact on molecular integrity. In-depth analysis of extraction parameters, such as solvent composition, temperature, and enzymatic exposure, allows for the generation of diverse reconstituted collagen forms, ranging from fibrous assemblies (fibrils and fibers) to isolated protein units (triple helices) and bioactive peptide fragments. Optimizing these parameters is crucial to balance yield, purity, and structural preservation.

A key phase in collagen extraction is enzymatic treatment, which gradually disassembles the dense fiber network into usable components [31,32]. A wide spectrum of enzymes has been employed, including trypsin, chymotrypsin, bromelain, and collagenase, each differing in cleavage sites and specificity [33]. Among those enzymes, pepsin remains the primary enzyme for biomedical-grade collagen extraction. Its effectiveness lies in selectively cleaving the non-helical telopeptide regions of collagen, while leaving the central triple-helical domain intact [34,35]. The selective cleavage is advantageous because it preserves the native fibrillar structure, enhances solubility, and maintains the biological activity of the extracted collagen. Furthermore, pepsin exhibits a higher affinity for unfolded proteins, allowing for greater recovery without excessive degradation into low-molecular-weight peptides [35]. This process prevents the undesirable disaggregation of collagen microfibrils or tropocollagen units, thereby preserving the mechanical and biofunctional properties essential for tissue engineering applications [33].

### 2.3. Artificial Intelligence (AI)-Based Tailoring and Optimization of Collagen

Tailoring collagen after extraction can be achieved through (1) crosslinking agents to improve mechanical strength and stability, (2) modification with bioactive molecules, including growth factors, to promote cell adhesion, proliferation, and differentiation, and (3) 3D bioprinting to create personalized collagen scaffolds with customized control over structure and properties [9,10,12]. Through these approaches, collagen can be formulated into diverse biomaterials, such as sponges, hydrogels, films, fibers, and injectable gels, providing versatile platforms for cell transplantation, wound healing, and regenerative medicine.

The transformative role of artificial intelligence (AI) in the processes of collagen extraction and optimization, including enhancing yield efficiency and reducing waste generation, was previously examined [36]. In this study, collagen valorization was aligned with biomedical principles, emphasizing transforming waste into high-value products, such as collagen peptides and biomaterials. As the cost of collagen is another obstacle, a comprehensive framework for cost-efficient collagen products that maximizes resource utilization can be achieved by integrating advanced AI technologies with diverse collagen resources [37].

Apart from process optimization, AI plays an essential role in the assessment of biological performance. One major goal in regenerative medicine is the induction of angiogenesis, which is critical for vascularizing engineered tissues and organoids [38]. Traditional preclinical studies often rely on animal models, but AI is now being harnessed to streamline this process.

For example, angiogenesis induced by collagen–polymer composite scaffolds was evaluated using the chorioallantoic membrane (CAM) model, where software designed for AI-enabled image analysis quantified blood vessel formation and vascular network complexity [39]. This study demonstrated the potential usage of the CAM model in combination with AI-based software for the evaluation of the mechanical performance of biomaterials, including 3D collagen–polymer composites.

The AI-based approach can be further evolved with Generative AI (GenAI) models, which are capable of designing massively generating composites, enabling the identification of stress distribution and producing improved mechanical properties that dictate stiffness and robustness of the material [40]. An AI model-based approach can efficiently utilize sparse data with limited resources and substitute for animal-based mechanical and therapeutic studies.

## 3. Application of Collagen Against Infectious Diseases

### 3.1. Major Role of Collagen in Infectious Diseases

Beyond its well-known role in supporting musculoskeletal tissues, maintaining tissue integrity, cellular function, and immune defense, collagen also fortifies the intestinal walls, skin fibroblasts, and keratinocytes, thereby forming both physical and biochemical barriers. These collagen-based structures regulate selective permeability; they facilitate the absorption of beneficial substances into the bloodstream while restricting the entry of harmful agents such as pro-inflammatory cytokines (IL-1β, IL-6, IL-8, TNF-α). In this way, collagen serves as a key mediator in sustaining tissue homeostasis and protecting against dysregulated immune responses.

The progression and severity of infectious diseases are strongly influenced by the dynamic regulation of collagen isoforms. Among the 28 collagen types identified in vertebrates, Type I and Type III have been particularly implicated in host–pathogen interactions and immune responses [41]. Dysregulation of these collagens, whether by overproduction, degradation, or structural alteration, can alter tissue susceptibility to infection and promote the inflammatory microenvironment. For example, excessive degradation of collagen during infection can weaken tissue barriers, facilitating pathogen invasion, while aberrant overproduction may contribute to fibrosis and chronic inflammation [42].

Collagen-related pathologies frequently arise from genetic defects [42] or nutritional deficiencies [43], which disrupt processes, such as biosynthesis, crosslinking, post-translational modification, or secretion. Mutations in collagen-coding genes can have wide-ranging consequences, including impaired skeletal and cartilage development, compromised skin barrier function, and muscular weakness. These findings suggest that collagen genes not only encode structural proteins but also act as critical regulators of developmental and homeostatic processes [43]. Notably, collagen synthesis requires at least eight distinct post-translational enzymes, several of which are now being explored as therapeutic targets. By modulating these enzymes, it may be possible to regulate collagen turnover and prevent pathological outcomes, such as excessive collagen accumulation in fibrotic diseases, which are frequently aggravated during or after chronic infections [42].

Emerging evidence also highlights the therapeutic potential of hydrolyzed collagen. Numerous in vitro studies have demonstrated that hydrolyzed collagen enhances the synthesis of pro-collagen-1α in skin fibroblasts and stimulates proliferation in both fibroblasts [44] and keratinocytes [45]. At the same time, it reduces the secretion of pro-inflammatory cytokines [46], which are major mediators in the initiation and maintenance of inflammatory responses.

Taken together, collagen’s numerous isoforms, structural diversity, and functional complexity place it in the position of a controller of normal organ physiology and pathological processes. Therefore, its modulation—through genetic, biochemical, or therapeutic means—represents both a challenge and an opportunity in advancing novel patient-specific treatment strategies.

### 3.2. Major Role of Collagen in Cardiovascular Diseases

Cardiovascular disease (CVD), particularly heart failure, remains the leading cause of mortality worldwide, excessively affecting elderly people and imposing a significant healthcare burden. At the core of cardiac tissue physiology lies a delicate balance between collagen synthesis and degradation, which governs tissue integrity, repair, and remodeling. Disruption of this equilibrium is essential to the progression of pathological remodeling displayed in cardiovascular diseases.

Following cardiac injury, collagen synthesis and deposition are stimulated by a network of molecular signals, including platelet-derived growth factor (PDGF), basic fibroblast growth factor (bFGF), transforming growth factor-β (TGF-β), interleukin-1 (IL-1), and tumor necrosis factor (TNF)-α [8,47]. These mediators activate fibroblasts, which in turn deposit extracellular matrix (ECM) components to reinforce the injured site. On the other hand, collagen degradation yields bioactive fragments—often termed *matrikines*—that stimulate fibroblast proliferation and induce the release of growth factors. These fragments not only modulate inflammation, but also promote angiogenesis and re-epithelialization, representing collagen’s dual role as both a structural and signaling molecule [48].

Despite advances in pharmacological treatments and interventional procedures, most existing therapies for heart failure remain temporary relief, focusing on symptom management rather than true myocardial regeneration. Strategies aimed at improving cardiac functions, such as infarct size reduction, angiogenic therapy, or cellular transplantation, have been widely explored, but with limited long-term efficacy. Following myocardial infarction, platelet activation and aggregation initiate fibrin clot formation at the injury site [49]. During the subsequent inflammatory phase, pro-inflammatory cytokines drive the migration of fibroblasts, epithelial cells, and endothelial cells to the injured myocardium, initiating a complex but often transient healing process [50].

One of the advanced therapies, a stem cell-based approach, once held great promise for myocardial repair, but clinical outcomes have revealed persistent limitations, including low cell retention and survival, reduced differentiation capacity, poor integration with the host myocardium, and immune rejection risks [21]. As a result, no current cellular therapy has achieved substantial functional regeneration of myocardial tissue. On the contrary, collagen functions as a critical mediator of cardiac repair—it regulates fibroblast proliferation and migration, directs differentiation pathways, and orchestrates ECM remodeling essential for structural recovery [21]. In this context, collagen-based biomaterials and scaffolds are an attractive choice for next-generation therapeutic platforms.

Advances in 3D-bioprinting technologies allow for the fabrication of personalized collagen scaffolds with controlled porosity, architecture, and biofunctionalization. These scaffolds can mimic the native cardiac ECM, providing a biologically relevant environment for transplanted cells and improving their retention, survival, and integration with host tissues. By combining structural support with the delivery of growth factors or stem cells, collagen scaffolds offer a promising strategy to overcome the shortcomings of conventional therapies, potentially accomplishing myocardial regeneration over transient repair.

In summary, collagen plays an active and multifaceted role in cardiovascular biology as a regulator of inflammation, angiogenesis, and tissue remodeling, while also serving as a foundation for innovative biomaterial-based interventions. At present, harnessing its biological complexity through engineered scaffolds and AI-assisted design approaches represents one of the most promising strategies in regenerative tissue engineering.

## 4. Hydrogel in 3D Printing and Tissue Regeneration

### 4.1. 3D Printing and Tissue Regeneration

Advances in biofabrication and platform technologies have profoundly transformed the biomedical field, allowing for not only accelerated preclinical drug development but also the rapid design and testing of vaccines and therapeutics against infectious diseases. Among these innovations, 3D bioprinting has emerged as one of the most versatile tools. Moreover, 3D bioprinting enables us to support personalization and customization, offering precise control over construct architecture, while integrating diverse therapeutic agents at variable doses, tailored to the physiological and pathological conditions of individual patients [51].

Over the past decade, remarkable progress in biocompatible materials, cell-friendly hydrogels, and supportive biomaterials has expanded the scope of 3D printing in regenerative medicine. Unlike conventional fabrication methods, 3D bioprinting provides spatial and temporal control over the deposition of biological materials, thereby reproducing the complex heterogeneity of native tissues and organs. The credentials of collagen are particularly valuable in engineering artificial organs, tissue patches, and bioactive scaffolds, where precision in geometry and composition determines functional outcomes.

Of particular significance are 3D cell-printing technologies that utilize live cell-laden hydrogels as “bioinks.” These systems enable the one-step fabrication of complete tissue constructs, where living cells are embedded within supportive matrices that mimic the natural extracellular environment [1,2,6]. Such constructs can function as in vitro disease models, facilitating the study of host–pathogen interactions, drug screening, and toxicology assays under physiologically relevant conditions. Alternatively, they can serve as implantable tissue substitutes designed to integrate with host systems and restore biological function.

Advances in printing resolution and bioink formulation now allow for the fabrication of vascularized constructs, addressing perennial problems in tissue engineering, namely, angiogenesis and nutrient perfusion in large tissue grafts. Furthermore, 3D printing enables iterative design and rapid prototyping, offering a cost- and time-efficient pathway for developing patient-specific solutions.

### 4.2. Natural Polymer Hydrogels as 3D-Printing Bioinks

Hydrogels are three-dimensional, water-swollen polymeric networks, exhibiting retentive properties for large volumes of water or biological fluids while maintaining structural integrity and high surface area [52,53,54]. Their hydrated environment and tunable mechanical properties make them highly suitable for supporting cell encapsulation and tissue-like constructs. Importantly, the physical and chemical composition of hydrogel matrices can be precisely modified to mimic the native extracellular matrix (ECM), thereby providing a biomimetic microenvironment that supports cellular adhesion, migration, and differentiation [55,56].

Bioinks, typically in hydrogel form, are used in 3D bioprinting to layer viable cells or therapeutic agents into functional structures [16,57]. Ideal hydrogel bioinks exhibit essential features, such as bioprintability, cell adhesion properties, insolubility in cell culture media, and biodegradability suitable for tissue regeneration [58]. Additional requirements include mechanical strength, rheological stability, and favorable chemical and biological properties—namely, non-toxicity and non-immunogenicity [59]. Artificially created materials have demonstrated higher mechanical properties, ease of manufacturing processes that are compatible with 3D printing, and easy and broad availability [3,60], whereas natural polymers, like collagen, are used more often in the biomedical field than synthetic resources due to their greater biological compatibility [61].

Among these materials, collagen-based hydrogels stand out as one of the most widely utilized natural bioinks. As previously mentioned, their fibrillar structure closely resembles the native ECM, promoting cell adhesion, proliferation, and differentiation while supporting tissue-specific remodeling. Moreover, the hybrid approach combines the versatile functionality of collagen with the mechanical robustness of synthetic polymers, yielding optimized bioinks suitable for diverse regenerative medicine applications.

### 4.3. Collagen-Based Hydrogels as 3D-Printing Bioinks

**Figure 2:**
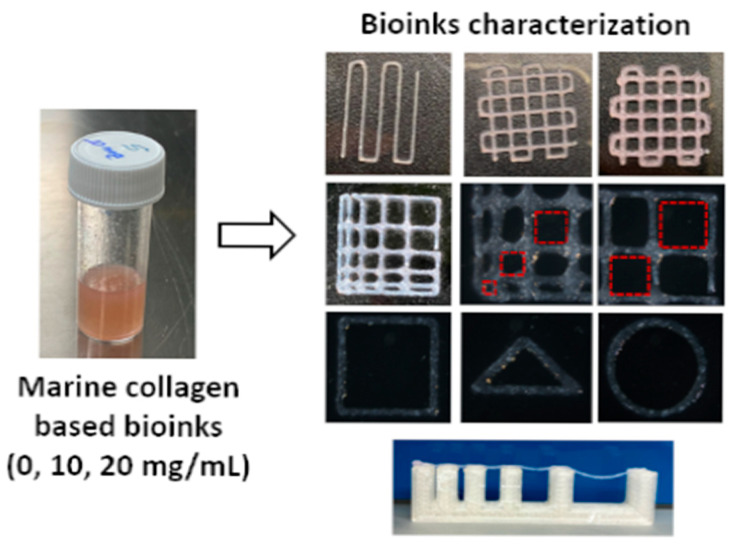
Marine collagen-based bioink for 3D bioprinting of a bilayered skin model [13] (cited from Cavallo et al., 2023).

Injectable collagen hydrogels have unique water-retaining capabilities, permeability to nutrients and metabolites, and intrinsic bioactivity, providing a biomimetic extracellular environment that fosters cell adhesion, migration, and differentiation while supporting the diffusion of oxygen, growth factors, and metabolic waste. As shown in Figure 2, collagen-based hydrogels play a pivotal role in regenerative medicine, particularly in contexts where rapid tissue repair and functional recovery are required.

A variety of fabrication strategies have been developed to produce collagen hydrogels with tunable structures and properties. Methods, such as salt-induced phase inversion [62], ultrasound-assisted formation [63], and molecular self-assembly [64], allow for precise control over fibril organization and crosslinking density. These approaches yield hydrogels that closely replicate the native fibrillar structure of collagen, thereby preserving its biological functions and enhancing regenerative potential [65]. The structural capability of these hydrogels is particularly advantageous in supporting tissue regeneration post-injury, where restatement of the native extracellular matrix (ECM) is critical.

Several preclinical studies delineated various biomedical applications and therapeutic influences of collagen composite hydrogels, as shown in Table 2. For instance, recombinant human collagen combined with chitosan has demonstrated enhanced efficacy in animal models of deep partial-thickness wounds, promoting angiogenesis, extracellular matrix remodeling, and accelerated vascular recovery [66,67]. Similarly, hydrogel composites of collagen I with hydroxybenzoic acid have proven to stimulate vascularization, epithelial growth, and organized collagen fiber deposition, thereby expediting wound closure and functional repair [68]. Another innovative approach involved incorporating collagen type I and hyaluronic acid into injectable hydrogel matrices, yielding an ECM-like environment that facilitates vascular-cell proliferation and tissue regeneration, while offering injectability for minimally invasive clinical applications [69].

In addition to native collagen, gelatin—a denatured and water-soluble derivative of collagen—has gained attraction as a complementary bioink. Gelatin supports robust cell adhesion and proliferation owing to the retention of Arg-Gly-Asp (RGD) peptide motifs from native collagen [70]. Although gelatin is thermosensitive—gelatin (at ~29 °C) and solubilizes near physiological temperature (~37 °C)—its gelling properties, tunability, and compatibility with chemical modification make it highly suitable for 3D-bioprinting applications [13,57]. Thus, gelatin is frequently used in hybrid hydrogels, where its printability is combined with collagen’s biofunctionality to yield scaffolds with both structural competence and biological performance.

**Table 2:** Collagen-containing hydrogels for 3D-bioprinting modalities and strategies.

Modality/Strategy	Strengths	Weakness	Applications	References
Extrusion bioprinting	Handles high-viscosity collagen blends	Lower resolution; shear stress	Lung epithelium models; vascular patches	[71]
Inkjet bioprinting	High throughput; multi-material	Requires low viscosity	Drug-screening microtissues	[58]
Laser-assisted (LAB)	High precision; gentle to cells	Cost, setup complexity	Patterned myocardium, Endothelial lattices	[14]
Light-based (SLA/DLP)	Fine features, rapid curing	Photo-initiator cytotoxicity	Microvasculature, cardiac valves	[14]
Collagen + chitosan	Printability, barrier mimicry	Batch variability	Lung tissue infection models	[71]
Collagen + hyaluronic acid	Angiogenesis, ECM-like	Needs crosslinking for strength	Cardiac/wound regeneration	[69]
Collagen + gelatin (GelMA)	Tunable gelation; cell adhesion	Thermosensitive	Myocardial patches, skin	[70]
Smart additives (e.g., black phosphorus)	4D stimuli-responsive, adaptive	Potential safety issue	4D bioinks for remodeling	[72]

### 4.4. Collagen in 3D Printing Against Infectious Diseases

Despite its biological superiority as a native extracellular matrix (ECM) component, collagen alone exhibits inherently low printability, limiting its direct use as a standalone bioink in 3D bioprinting [11]. Its modest rheological properties, weak mechanical stability, and rapid degradation make it inappropriate for producing durable, high-capability constructs. To overcome these challenges, collagen is frequently blended with complementary biomaterials, chemically functionalized, or reinforced through crosslinking agents. These strategies significantly improve collagen’s stability and printability, enabling its integration into biomimetic models for tissue engineering and regenerative medicine [73,74].

To ensure clinical relevance, particularly in applications requiring long-term stability, collagen-based scaffolds are typically subjected to crosslinking treatments. Crosslinking improves mechanical integrity, enzymatic resistance, and degradation control, all of which are essential for maintaining scaffold functionality in vivo [75,76]. The choice of crosslinking method—whether chemical (e.g., glutaraldehyde, carbodiimide), physical (e.g., UV irradiation, dehydrothermal treatment), or enzymatic (e.g., transglutaminase)—directly influences scaffold durability, biocompatibility, and immunogenicity. Hybrid collagen formulations incorporating natural molecules (e.g., glycosaminoglycans, tricalcium phosphates) or synthetic polymers (e.g., polyglycerol methacrylate) have further improved mechanical performance and biofunctionality [70]. Similarly, grafting collagen scaffolds with cellulose, alginate, chitosan, or gelatin derivatives has been shown to preserve structural rigidity and expand functional versatility [73,77,78].

A particularly promising approach involves collagen-based hydrogel composites. For example, collagen–chitosan hydrogels have been employed as printable scaffolds to engineer lung tissue models for infectious disease research [71]. These scaffolds not only demonstrated excellent printability, but also supported high cell viability and recapitulated epithelial morphology, providing physiologically relevant in vitro models for studying host–pathogen interactions [79]. Likewise, commercially available formulations, such as the LifeSupport^®^ gelatin suspension (Advanced Biomatrix) and alginate/gelatin-based bioinks, have already been successfully used to fabricate diverse constructs, including liver models, nasal cartilage, heart valves, and entire bio-printed hearts [80]. These examples highlight the translational potential of collagen-containing bioinks in building both disease models and implantable tissues.

The incorporation of intelligent and programmable elements within collagen-based scaffolds has recently attracted surmounting attention, leading to 4D bioprinting. One such example is the addition of black phosphorus nanosheets, which allow bioinks for enhanced mechanical adaptability, biodegradability, and bioactivity [72]. These smart composites can respond dynamically to physiological stimuli, enabling more versatile, bioinspired scaffolds that are capable of addressing the complex demands of tissue engineering and infectious disease modeling.

### 4.5. Collagen in 3D Printing Against Cardiovascular Diseases

Persistent challenges are still present in the delivery of biocompatible materials and functional cells for cardiovascular disease interventions, particularly in ensuring (1) sufficient delivery of myocytes or progenitor cells, (2) effective retention and integration of these cells with adequate nutritional support, and (3) the identification of biomaterials that positively influence cardiac tissue contraction [81].

To address these issues, 3D bioprinting has been utilized in fabricating myocardial tissue using a variety of biomaterials, including polycaprolactone (PCL), sodium alginate, gelatin/methylbenzene sulfonate (MBS) extracellular matrix scaffolds, and decellularized ECM. These scaffolds are often designed through extrusion-based printing techniques to replicate cardiac microenvironments. However, the application of such biomaterials introduces concerns regarding immunogenicity, host inflammatory reactions, fibrotic encapsulation, degradation kinetics, and the toxicity resulting from byproducts—all of which compromise long-term functional integration of engineered tissues [81].

Recent investigational studies have displayed the viability of collagen-containing bioprinted constructs for cardiac tissue repair. Collagen–gelatin composites have been employed to fabricate myocardial patches with enhanced mechanical strength and improved electrical conductivity when combined with conductive nanomaterials [22]. Similarly, collagen–hyaluronic acid hydrogels have been shown to foster angiogenesis and neovascularization, essential for sustaining engineered cardiac tissues [82]. By incorporating growth factors or vascular cells directly into collagen-based scaffolds, researchers have reported improved cell retention and survival, addressing one of the major limitations of stem cell therapy.

### 4.6. Bioavailability of the 3D-Printed Collagen Products

The performance of 3D-printed collagen depends on the collagen concentration in the bioink, which should be greater than 20 mg/mL to achieve the intended reproducibility of 3D printing [83,84]. At present, a few commercially available collagen bioinks, such as Lifeink^®^ (35 mg/mL, Advanced Biomatrix, Carlsbad, CA, USA) and Viscoll^®^ (80 mg/mL, Imtek, Moscow, Russia), meet those requirements [83,85].

The metabolic products of 3D-printed collagen-based products are amino acids (i.e., the major components of collagen), which are released as they are degraded in the body [27]. This process is mostly the same as the body’s natural metabolic process of collagen, which usually does not cause any immunogenic responses or cytotoxicity to the body, but its metabolic rate is determined by the properties of the bioink resources and the surrounding environmental conditions, including enzyme activity and host responses, such as interaction with endogenous components and fibrin clot formation [85].

## 5. Challenges and Future Directions

Since the onset of the COVID-19 pandemic, the biomedical field has been challenged to accelerate the development of vaccines and therapeutics capable of preventing pathogen spread and mitigating global health crises [4]. Traditional strategies, though effective, often require extended timelines for preclinical testing, regulatory approval, and large-scale manufacturing. In contrast, 3D bioprinting has demonstrated its potential to provide rapid, customizable, and scalable therapeutic solutions, thereby reshaping the status of preventive and regenerative medicine.

Within the realm of preventive medicine, 3D printing with collagen-based bioinks offers unique opportunities to fabricate physiologically relevant models for screening vaccine and drug candidates. Collagen scaffolds can be engineered to regenerate complex tissues with a hierarchical architecture that closely resembles native biological systems. The conformity of these structures has been enhanced by recent advances in collagen modification processes, including chemical crosslinking and blending with exogenous materials. These modifications allow printed products to achieve greater structural stability and tunable degradation, and more closely simulate the biomimicry of native extracellular matrices.

The combination of modern 3D manufacturing technologies with traditional or advanced computational tools, including artificial intelligence (AI) and quantum computing (QC), has ushered in a new era for tissue engineering and regenerative medicine. AI can be employed to optimize parameters, such as printing resolution, bioink viscosity, and crosslinking density, ensuring high reproducibility and enhanced cell viability. Moreover, AI-driven predictive modeling enables the design of patient-specific prototypes, tailored to biological variability and clinical requirements. QC, though still in its infancy for biomedical applications, holds promise for accelerating molecular simulations and protein–material interaction studies, which could revolutionize the customization of collagen-based bioinks [86,87].

Collagen-based 3D bioprinted constructs, when paired with AI-powered analysis platforms, are already proving to be highly effective for in vitro disease modeling. Applications in cardiovascular diseases and infectious disease modeling have shown that collagen-based 3D models could provide reproducible platforms for preclinical assessment, reduce reliance on animal testing, streamline translational pipelines, personalize treatment, and support more rapid clinical deployment of preventive and therapeutic measures.

In summary, the convergence of collagen bioinks, 3D printing, and AI-driven computational technologies is setting the stage for transformative progress in preventive and regenerative medicine, which can be customized according to patients’ status. Despite these advances, ongoing challenges, including the inherent limitations in printability, stability, and cell viability, as well as the mechanical weakness of collagen-based bioinks, necessitate further refinement through hybridization with synthetic polymers, nanomaterials, or smart functional additives designed for 4D printing. The addition of nanosensors that allow for real-time, continuous monitoring of biological fluids to which collagen-based bioinks are exposed can also be implemented. Nonetheless, collagen-based 3D constructs will become an invaluable asset for the prevention and treatment of infectious and cardiovascular diseases.

## Figures and Tables

**Table 1:** Collagen resources for biomedical use and 3D-bioprinting bioinks.

Source	Dominant Type(s)	Key Attributes	Extraction Method	Pros/Cons	Major Usages	References
Bovine/Porcine Skin	I, III	Random fiber orientation; moderate strength	Acid-soluble or pepsin-assisted	Pros: readily available; cons: fast degradation, fibrillogenic	Wound dressings, Dermal scaffolds	[8,12,13,21,22]
Bovine/Porcine Tendon	I	Residual packing; high tensile strength	Pepsin-assisted	Pros: mechanical robustness; cons: sourcing issues	Load-bearing scaffolds, surgical meshes	[23]
Equine Tendon	I	High purity; slow degradation	Pepsin-assisted	Pros: biocompatibility; cons: limited supply	Regenerative implants, grafts	[24,25]
Fish Skin/Scales	I	Lower temp denaturation; bioactive peptides	Acid or mild enzymes	Pros: sustainable, by-product use; cons: lower strength, antibacterial gap	Wound care, nutraceuticals	[26]
Recombinant/Engineered	I variants	Tunable, batch consistency	Microbial/plant expression	Pros: customizable; cons: cost, yield	Advanced bioinks, disease models	[12,13,27]

## Data Availability

No new data were created or analyzed in this study. Data sharing is not applicable to this article.

## References

[1] Duval K., Grover H., Han L.H., Mou Y., Pegoraro A.F., Fredberg J., Chen Z. (2017). Modeling physiological events in 2D vs. 3D cell culture. Physiology.

[2] Veerubhotla R., Clark J. (2021). 3D printing for regenerative medicine: Technologies and tools for clinical translation. Expert Opin. Biol. Ther..

[3] Veerubhotla R., Tandon B., Clark J. (2022). Role of artificial intelligence and quantum computing in bioengineering: Advances and future applications. Biogen. Transl. Med..

[4] Yi C., Sun X., Lin Y., Gu C., Ding L., Lu X., Fan H. (2021). COVID-19: What has been learned and to be learned about the novel coronavirus disease. Int. J. Biol. Sci..

[5] Lee W., Debasitis J.C., Lee V.K., Lee J.H., Fischer K., Edminster K., Yoo S.S. (2016). Multi-layered culture of human skin fibroblasts and keratinocytes through three-dimensional freeform fabrication. Biomaterials.

[6] Zimmerling A., Chen X. (2020). 3D bioprinting for skin tissue engineering: Current status and perspectives. J. Tissue Eng..

[7] Lee C.H., Singla A., Lee Y. (2001). Biomedical applications of collagen. Int. J. Pharm..

[8] Chattopadhyay S., Raines R.T. (2014). Collagen-based biomaterials for wound healing. Biopolymers.

[9] Gómez-Guillén M.C., Giménez B., López-Caballero M.E., Montero M.P. (2011). Functional and bioactive properties of collagen and gelatin from alternative sources: A review. Food Hydrocoll..

[10] Marques C.F., Diogo G.S., Pina S., Oliveira J.M., Reis R.L. (2019). Collagen-based bioinks for hard tissue engineering applications: A comprehensive review. J. Mater. Sci. Mater. Med..

[11] Debnath S., Bhowmick S., Das A. (2025). Emerging trends in collagen-based biomaterials for soft and hard tissue engineering. Tissue Eng. Regen. Med..

[12] Wang X., Zhang Y., He J., Liu M., Li J. (2023). Recent advances of collagen-based bioinks in 3D bioprinting. Biomater. Sci..

[13] Cavallo C., Desando G., Martini L., Roffi A., Zini N., Bartolotti I., Grigolo B. (2023). Collagen-based bioinks for 3D bioprinting: Advances and applications in regenerative medicine. J. Clin. Med..

[14] Vidler T., Jayasuriya S., Atif A. (2024). Light-assisted 3D printing technologies for biomedical applications: A review. Biofabrication.

[15] Advanced Biomatrix Report. https://advancedbiomatrix.com.

[16] Stepanovska J., Stachurova T., Raska M. (2021). Bioink development for 3D bioprinting: A biomaterial perspective. Materials.

[17] Allan J.T., Watt F.M. (2001). Influence of extracellular matrix composition and organization on cell shape, cytoskeleton, and adhesion. Nat. Cell Biol..

[18] Long C., Li X., Zhang Y., Zhao Y. (2015). Self-assembly of Type I collagen: Morphology regulation, molecular packing, and growth mechanism. Biomacromolecules.

[19] Gauza-Wlodarczyk M., Kubisz L., Wlodarczyk D. (2017). Amino acid composition in determining collagen type for the production of biomedical materials. Adv. Clin. Exp. Med..

[20] Drago D., Cossetti C., Iraci N., Gaude E., Musco G., Pluchino S. (2014). The stem cell secretome and its role in brain repair. Biochimie.

[21] Brett D. (2008). A review of collagen and collagen-based wound dressings. Wounds.

[22] Liu T., Hao Y., Zhang Z., Zhou H., Peng S., Zhang D., Li K., Chen Y., Chen M. (2024). Advanced Cardiac Patches for the Treatment of Myocardial Infarction. Circulation.

[23] Ponugoti N., Xu F., Nguyen K.C., Zhou Q., Zhang X. (2013). Mechanical characterization of collagen-based scaffolds prepared from tendon and skin. Mater. Sci. Eng. C.

[24] Coraca-Huber D.C., Hausdorfer J., Fille M., Steidl M., Nogler M. (2014). Collagen from equine tendon for application in medicine and tissue engineering. Sci. World J..

[25] Moon H.J., Ko D.Y., Kim J., Jung M., Min S.K., Kim J.H. (2015). Physical properties and biocompatibility of horse tendon collagen as a potential scaffold for tissue engineering. Biotechnol. Bioprocess Eng..

[26] Gaikwad K.K., Kim M. (2024). Fish-derived collagen and its role in regenerative medicine: Recent insights and future directions. Mar. Drugs.

[27] Wang Z., Ma D., Liu J., Xu S., Qiu F., Hu L., Liu Y., Ke C., Ruan C. (2025). 4D printing polymeric biomaterials for adaptive tissue regeneration. Bioact. Mater..

[28] Sakai S., Kawakami K., Hirose M. (2015). Production of collagen scaffolds from tendon and their properties. J. Biomater. Appl..

[29] Zhou Q., Lu Y. (2016). Structural differences in collagen from various mammalian tissues and their implications for biomedical use. Biomed. Mater..

[30] Liu J., Xie M., Gao Y., Chen X. (2025). Comparative analysis of marine and mammalian collagens: Mechanical properties and antibacterial performance. J. Biomed. Mater. Res. A.

[31] Riley G.P., Herman S.M. (2005). Extraction and solubilization of collagen from tendon tissue. Matrix Biol..

[32] Zhao Y., Zhang K., Wang J. (2012). Enzymatic degradation of collagen: An insight into collagenase activity. J. Enzyme Inhib. Med. Chem..

[33] Franciosi E., Alessandri S., Mari A., Tursi A. (2007). Enzyme-assisted extraction and characterization of collagen. Int. J. Biol. Macromol..

[34] Matsushita O., Koide T., Kobayashi R., Nagata K., Okabe A., Maeda H. (1994). Substrate recognition and cleavage site of Clostridium histolyticum class I collagenase. J. Biol. Chem..

[35] Rawlings N.D., Barrett A.J. (1995). Evolutionary families of metallopeptidases. Methods Enzymol..

[36] Srinivasan R., Kim S., Gupta R. (2025). Artificial intelligence–driven optimization of collagen valorization from food and biomedical waste. Biomater. Adv..

[37] Li J., Zhang Y., Zhang W., Liu H. (2021). Angiogenesis in tissue engineering: From mechanistic understanding to advanced biofabrication. Adv. Healthc. Mater..

[38] Harder D.R. (2023). AI in biomedical modeling: From mechanistic pathways to personalized medicine. Nat. Rev. Bioeng..

[39] Salvante A., Giacobazzi R., Martini L., Candiani G. (2024). AI-assisted angiogenesis quantification on the CAM assay for scaffold evaluation. Mater. Sci. Eng. C.

[40] Masrouri M., Qin Z. (2024). Towards data-efficient mechanical design of bicontinuous composites using generative AI. Theor. Appl. Mech. Lett..

[41] Singh A., Dwivedi S., Srivastava R., Verma P. (2023). Collagen in infectious diseases: From barrier function to immune modulation. Front. Immunol..

[42] Myllyharju J., Kivirikko K.I. (2001). Collagens, modifying enzymes, and their mutations in humans, flies, and worms. Trends Genet..

[43] Arseni L., Lombardi A., Orioli D. (2018). From collagen biosynthesis to collagenopathies: Challenges and opportunities. J. Biol. Chem..

[44] Zaarour B., Younes I., Rinaudo M. (2022). Bioactive effects of hydrolyzed collagen on skin fibroblast proliferation. Mar. Drugs.

[45] Ortiz-López M., Peredo-Escárcega A., de Jesús Delgado-López D. (2022). The impact of marine-derived collagen on human keratinocyte function and wound healing. Tissue Eng. Part A.

[46] Brandao-Rangel E., Nascimento J.A., Cruz M.S. (2022). Anti-inflammatory properties of hydrolyzed collagen peptides in human cell cultures. Biomed. Pharmacother..

[47] Wareham L.K., Baratta R.O., Del Buono B.J., Schlumpf E., Calkins D.J. (2024). Collagen in the central nervous system: Contributions to neurodegeneration and promise as a therapeutic target. Mol. Neurodegener..

[48] Mathew-Steiner S.S., Roy S., Sen C.K. (2021). Collagen fragments and wound healing: From bench to bedside. Adv. Wound Care.

[49] Tronci G., Russell S.J., Wood D.J. (2019). Biomimetic approaches to designing collagen-based cardiovascular scaffolds. Front. Bioeng. Biotechnol..

[50] Harsha V.K., Brundha M.P. (2020). Inflammatory mechanisms in cardiac tissue remodeling: A histopathological review. Res. J. Pharm. Technol..

[51] Konta A.A., García-Piña M., Serrano D.R. (2017). Personalised 3D printed medicines: Which techniques and polymers are more successful?. Bioengineering.

[52] Ahmed E.M. (2015). Hydrogel: Preparation, characterization, and applications: A review. J. Adv. Res..

[53] Chai Q., Jiao Y., Yu X. (2017). Hydrogels for biomedical applications: Their characteristics and the mechanisms behind them. Gels.

[54] Li J., Mooney D.J. (2022). Designing hydrogels for controlled drug delivery. Nat. Rev. Mater..

[55] Fan C., Cui F.Z., Zhang H.Y. (2021). Functionalized natural hydrogels for tissue engineering. Adv. Healthc. Mater..

[56] Ho C.M., Ng S.H., Li K.H.H., Yoon Y.J. (2022). 3D printed hydrogels for tissue engineering applications. Adv. Drug Deliv. Rev..

[57] Moss E.A., Nanduri M., Collins M.N. (2025). Hydrogel-based bioinks for 3D bioprinting in regenerative medicine. Adv. Mater. Interfaces.

[58] Hospodiuk M., Dey M., Sosnoski D., Ozbolat I.T. (2017). The bioink: A comprehensive review on bioprintable materials. Biotechnol. Adv..

[59] Lee J.M., Sing S.L., Zhou M., Yeong W.Y. (2015). 3D bioprinting processes: A perspective on classification and terminology. Int. J. Bioprinting.

[60] Abelardo E., Thomas D.J., Jessop Z.M., Whitaker I.S. (2018). Synthetic material bioinks. 3D Bioprinting for Reconstructive Surgery.

[61] Veerubhotla S., Lee Y. (2022). Smart collagen–alginate hydrogel: Fabrication and biomedical applications. Bioengineering.

[62] Wong K.H.K., Chan J.M.W., Lo A.C.Y. (2011). Fabrication of collagen-based hydrogel using salt-induced phase inversion for tissue engineering applications. Mater. Sci. Eng. C.

[63] Norris K., Alvarez Z., Matson J.B. (2019). Ultrasound-assisted formation of injectable collagen-based hydrogels for localized therapy. ACS Biomater. Sci. Eng..

[64] Jiang T., Xu G., Wang Q., Pan H. (2019). Molecular self-assembly of collagen-mimetic hydrogels with tunable mechanical properties. Biomacromolecules.

[65] Islam M.M., Fukunaga Y., Sato M. (2015). Fabrication of biomimetic collagen scaffold and its application in tissue engineering. J. Biomed. Mater. Res. A.

[66] Deng C., Li X., Zhang L. (2021). Injectable hydrogel with enhanced angiogenesis and tissue remodeling for burn wound healing. Biomater. Sci..

[67] Jridi M., Abdelhedi O., Nasri M. (2015). Composite hydrogel from fish skin collagen and polysaccharides: Application in wound healing. Colloids Surf. B.

[68] Ying Y., Liu Y., Wu Y. (2019). Collagen I-hydroxybenzoic acid hydrogel enhances vasculature and epithelial regeneration. ACS Omega.

[69] Gao Y., Zhu Y., Zhang Y. (2018). Injectable collagen–hyaluronic acid hydrogels for wound healing. Acta Biomater..

[70] Kim B.S., Kim H., Gao G., Cho D.W. (2016). 3D bioprinting of functional tissue constructs using bioink based on gelatin and collagen. Biofabrication.

[71] Suo H., Wang Y., Chen G. (2021). Collagen–chitosan hydrogel scaffolds for 3D printing of lung tissue constructs. Mater. Sci. Eng. C.

[72] Bai Z., Liu W., Wang Y. (2024). Intelligent black phosphorus–collagen bioink for smart tissue regeneration. Adv. Funct. Mater..

[73] Yin J., Yan M., Wang Y., Fu J., Suo H. (2018). 3D printing of low concentration alginate-based scaffolds for cell expansion and transplantation. Biofabrication.

[74] Shin J.H., Kang H.W. (2018). The development of gelatin-based bio-ink for use in 3D hybrid bioprinting. Int. J. Precis. Eng. Manuf..

[75] Delgado L.M., Bayon Y., Pandit A., Zeugolis D.I. (2015). In vitro biological characterization of collagen scaffolds. J. Biomed. Mater. Res. B.

[76] Adamiak K., Sionkowska A. (2020). Current methods of collagen crosslinking: Review. Int. J. Biol. Macromol..

[77] Fullana S.G., Torres-Giner S., Lagaron J.M. (2012). Improved collagen nanofibers through blending with natural polysaccharides. Carbohydr. Polym..

[78] Zhang Y.S., Khademhosseini A. (2015). Advances in engineering hydrogels. Science.

[79] Bhowmick S., Dinda A.K., Mandal T.K. (2018). Lung tissue engineering: Recent developments and future prospects. Adv. Healthc. Mater..

[80] Hiller M., von der Helm C., Behr J.M. (2018). Development of ready-to-use bioinks for diverse 3D bioprinting applications. Biofabrication.

[81] Hibino N., Duncan D.R., Nalbandian A., Shinoka T. (2017). Challenges to myocardial tissue engineering: Cell delivery, biomaterial selection, and immunogenicity. Circ. J..

[82] Wei Z., Lei M., Wang Y. (2023). Hydrogels with tunable mechanical plasticity regulate endothelial cell outgrowth in vasculogenesis and angiogenesis. Nat. Commun..

[83] Isaeva E.V., Beketov E.E., Demyashkin G.A., Yakovleva N.D., Arguchinskaya N.V., Kisel A.A. (2022). Cartilage formation in vivo using high concentration collagen-based bioink with MSC and decellularized ECM granules. Int. J. Mol. Sci..

[84] Li Z., Ruan C., Niu X. (2023). Collagen-based bioinks for regenerative medicine: Fabrication, application and prospective. Med. Nov. Technol. Devices.

[85] Osidak E.O., Kozhukhov V.I., Osidak M.S., Domogatsky S.P. (2020). Collagen as Bioink for Bioprinting: A Comprehensive Review. Int. J. Bioprint.

[86] Faruque A.V., Alam S., Biswas K. (2023). Artificial intelligence-enhanced 3D bioprinting of collagen scaffolds for regenerative medicine. J. Biomed. Mater. Res. A.

[87] Shelke N.B., James R., Laurencin C.T. (2024). AI-assisted control in collagen-based biofabrication: A pathway to real-time optimization. Adv. Healthc. Mater..

